# miR-652 Inhibits the Proliferation, Migration, and Invasion of Osteosarcoma Cells by Targeting HOXA9 and Regulating the PI3K/Akt Signaling Pathway

**DOI:** 10.1155/2022/4809312

**Published:** 2022-01-24

**Authors:** Chao Yang, Yang Chen, Wenhua Xiong, Ke Xu

**Affiliations:** Department of Orthopaedics, Hwa Mei Hospital, University of Chinese Academy of Sciences, Ningbo Institute of Life and Health Industry, Ningbo 315010, Zhejiang, China

## Abstract

**Objective:**

The aim of this study was to investigate the abnormal expression of miR-652 in osteosarcoma and its related mechanism.

**Materials and Methods:**

Reverse transcription-polymerase chain reaction (RT-PCR) was used to detect the expression of miR-652 and HOXA9 in osteosarcoma tissues and normal tissues. A bioinformatics method was used to predict target genes of miR-652, and then luciferase reporter genes and western blot tests were used to verify expression of target genes. The miR-652 overexpression models were established by transfecting miR-652 mimics into osteosarcoma U-2OS cells, and HOXA9 overexpression models were simultaneously established by transfecting pcDNA3.1-HOXA9 into osteosarcoma U-2OS cells. Cell proliferation ability was detected by the CCK-8 assay, cell migration ability was detected by the scratch test, and cell invasion ability was detected by the Transwell invasion assay. Western blot tests were used to verify the expression of HOXA9, p-PI3K, p-AKT, MMP2 and MMP9.

**Results:**

miR-652 and HOXA9 showed low expression and overexpression, respectively, in osteosarcoma tissues. Proliferation, invasion, and migration abilities of osteosarcoma cells and the level of protein expression of p-PI3K, p-Akt, MMP2, and MMP9 were significantly decreased with enhanced miR-652 expression (*P* < 0.01), while overexpression of HOXA9 reversed this situation. The results of dual-luciferase reporter gene showed that expression and activity of HOXA9 were downregulated accordingly, and the level of HOXA9 protein was decreased with enhancing miR-652 expression (*P* < 0.01).

**Conclusion:**

miR-652 may negatively regulate HOXA9 expression and inhibit the proliferation, migration, and invasion abilities of osteosarcoma cells through the PI3K/Akt signaling pathway.

## 1. Introduction

Osteosarcoma (OS) is a highly malignant osteogenic tumor with a high rate of metastasis and recurrence. The total incidence of osteosarcoma in the population is about 2∼3 million every year, which mainly occurs in teenagers. The incidence of osteosarcoma in males is about 1.4 times higher than that of females. The prognosis of osteosarcoma is poor because of the strong ability of invasion and metastasis, which seriously affects the quality of life of patients [1]. Therefore, looking for tumor markers of osteosarcoma to help with its early diagnosis and treatment has become the focus of current research.

MicroRNAs (miRNAs) are a class of endogenous noncoding small RNA molecules with a length of about 18,022 nucleotides, which mainly regulate the expression of downstream target genes by directly interacting with the 3′UTR region of the target gene mRNA [2]. A large number of studies have confirmed that miRNAs are involved in a variety of biological processes, such as cell proliferation [3], apoptosis [4], differentiation [5], migration [6], and invasion [7]. It has been proven that miRNA has direct or indirect effects on osteosarcoma, such as targeting matrix metalloproteinase 13 (MMP13) and B cell CLL/lymphoma 2 (Bcl-2). miR-143 can be involved in the lung metastasis of human osteosarcoma cells, which can be used as a target for tumor therapy [8, 9]. In addition, downregulation of miR-199a-3p may accelerate the growth and proliferation of osteosarcoma cells; the restoration of miR-199a-3p expression may be helpful to the treatment of osteosarcoma [1]. Some studies have suggested that miR-34a is an effective gene therapy, which can inhibit the lung metastasis of osteosarcoma by regulating c-Met and other genes and play the role of tumor suppressor genes [2]. Previous studies have reported that the expression of miR-652 is abnormally low in the tissues of patients with osteosarcoma [10], suggesting that it may be involved in the occurrence and development of osteosarcoma, but the specific regulatory mechanism is not clear. The purpose of this study is to reveal the role of miR-652 in osteosarcoma and its related molecular mechanism from the perspective of cell invasion and migration.

HOXA9 is a member of the family A homeobox (HOX) gene [8], which is composed of exon CD with a splicing site and exon II that encodes a DNA structural region. Its gene promoter region contains rich CPG islands and encodes transcriptional regulators with a classical helix-corner-helix structure. As an important transcriptional regulatory factor, HOXA9 participates in tumorigenesis and development [1, 8, 9]. Abnormal expression of HOXA9 in malignant tumors can regulate not only cell proliferation and apoptosis, cell differentiation, invasion and metastasis, and drug resistance, but also the proliferation and self-renewal of tumor stem cells [1, 9]. It is expected to become an index for tumor diagnosis, treatment, and prognosis evaluation [2]. However, the specific role and mechanism of HOXA9 in osteosarcoma is not clear.

The purpose of this study was to investigate the expression level of miR-652 in osteosarcoma and the effect of miR-652 on the biological characteristics of osteosarcoma cells and its mechanism. At the same time, the regulatory effect of miR-652 on HOXA9 was analyzed in order to provide a new idea for gene-targeted therapy of osteosarcoma.

## 2. Materials and Methods

### 2.1. Collection of Clinical Specimens

Thirty osteosarcoma tissue samples obtained from osteosarcoma surgery in our hospital were collected as the experimental group. All the patients were treated for the first time without chemotherapy. The pathological types of osteosarcoma were determined independently by 2 pathologists. The tumor length was 1.4–6 cm, with an average length of 4.14 cm. The mean age of patients was (22 ± 4.2) years. All operations were radical resections. 30 normal bone tissue specimens who underwent amputation at the same time were collected as the control group. There were no statistically significant differences in gender or age between the two groups. All patients signed informed consent forms. This study was approved by the Ethics Committee of Hwa Mei Hospital.

### 2.2. Cell Culture

Normal osteoblasts (hFOB 1.19) and OS cell lines (HOS, U2OS, SJSA1, Saos2, MG63) were purchased from the Cell Bank of the Shanghai Chinese Academy of Sciences. All cells were incubated in RPMI-1640 medium (containing 10% fetal bovine serum and 1% penicillin-streptomycin) for 72 hours at 37°C with 5% CO_2_, and then the cells were digested with 0.25% trypsin for passage when the cells converged to about 90%.

### 2.3. Experimental Grouping and Transfection

HOS and MG63 in the logarithmic phase were selected and divided into four groups: the blank control group, the miR-NC group, the miR-652 mimics group, and the pcDNA3.1-HOXA9 + miR-652 mimics group, according to the experimental design. The blank control group did not receive any treatment, and the plasmids of miR-NC mimics, miR-652 mimics, and pcDNA3.1-HOXA9 were transfected into U-2OS cells, respectively. The specific operations were carried out according to the Lipofectamine TM 2000 instructions (Life Technologies, Rockville, MD, USA).

### 2.4. CCK-8 Assay

HOS and MG63 were inoculated on 96-well plates at a concentration of 5 × 10^4^ cells per well when successfully transfected. At 0, 12, 24, and 48 h after transfection, 10 *μ*L CCK-8 reagent and 90 *μ*L medium were added to each well, and the cells were incubated for 2 h. The absorbance of each well was measured by automatic enzyme labeling (Tecan Sunrise, Austria) at a 450 nm wavelength. Taking the control group as a reference, the ratio of A value of the other groups to the control group was calculated as the proliferation rate, and the experiment was repeated 3 times.

### 2.5. Transwell Assay

The U-2OS cells were digested with 0.25% trypsin when successfully transfected and washed with PBS. The cell density was adjusted to 5 × 10^5^/mL by resuspension of DMEM serum-free medium. 50 *μ*L diluted Matrigel (diluted at 1 : 8 with serum-free DMEM medium) was added to the upper layer of the Transwell chamber, and the Matrigel was solidified overnight at 37°C. After the cells were digested, washed, and resuspended in the serum-free medium, 200 *μ*L cell suspension was added to the upper chamber of the Transwell, and the medium containing 10% FBS was added to the 24-well plate. In the incubator at 37°C and 5%CO_2_ for 48 h, the chamber was taken out when the cells were incubated at 37°C and 5% CO_2_ for 48 h. The cells were fixed with 95% ethanol for 10 min, and then stained with 0.1% crystal violet for 15 min, finally washed with PBS. The number of cells passing through the membrane was directly observed by an inverted microscope and photos were taken.

### 2.6. RT-qPCR Assay

According to the instructions of the TRIzol kit, the total RNA of osteosarcoma tissues and U-2OS cells was extracted. The concentration and purity of RNA were determined and calculated by a spectrophotometer. When D260 nm/D280 nm was 1.8–2.1, it was a qualified RNA sample. At the same time, 2% denaturing agarose gel electrophoresis was performed to evaluate the integrity of RNA. The qualified RNA samples were reverse-transcribed into cDNA by an RNA reverse transcription kit, and the cDNA was used as a template for quantitative PCR detection. The 2^−△△Ct^ relative quantitative method was used to detect the expression level of miR-652 and the target gene HOXA9 in each group.

### 2.7. Western Blot

U-2OS cells were lysed with RIPA lysate after being transfected for 48 h, and the total protein was extracted. The protein concentration was determined by the BCA method. 40 *μ*g of protein was taken from each group and added to the loading buffer. The protein was denaturated at 95°C for 10 min, electrophoresis was carried out, and the membrane was transferred (Millipore, Bedford, MA, USA). 5% skimmed milk was sealed at room temperature for 1 h, and primary antibodies (HOXA9, MMP-2, and MMP-9) were incubated at 4°C overnight. The HRP-labeled secondary antibody was incubated at room temperature for 2 h. The images were then taken with a gel imaging system after adding a chemical illuminator.

### 2.8. Dual-Luciferase Reporter Gene Assay

The cells were inoculated into 12-well plates after 24 h of transfection and divided into the wild-type plasmid group, the mutant plasmid group, and the control group. The mutant plasmid group was cotransfected with miR-652 mimics and mutant luciferase plasmid pGL3-HOXA9-3 luciferase plasmid, the wild-type plasmid group was cotransfected with miR-652 mimics and wild-type luciferase plasmid pGL3-HOXA9-3 luciferase plasmid, and the control group was cotransfected with miR-652 mimics and control luciferase plasmid. The fluorescence activity was detected by the luciferase reporter gene system after being transfected for 24 h. The experiment was repeated 3 times. After being mixed well, the relative light unit (RLU) was detected by the fluorescence meter. After an interval of 10 min, 100 *μ*L of Renilla luciferase detection reagents were added, and the RLU of the internal reference plasmid pRL-TK was determined after mixing, and relative luciferase activity was calculated.

### 2.9. Statistical Analysis

All the data were analyzed by SPSS 22.0 software (SPSS Inc., Chicago, IL, USA), and the data were expressed by mean ± SD. The unpaired Student's *t*-test was used to compare the mean values of the two groups, and the ANOVA test was used to compare the mean values of multiple groups.

## 3. Results

### 3.1. The Expression of miR-652 in Osteosarcoma Tissues and Cell Lines

**Figure 1:**
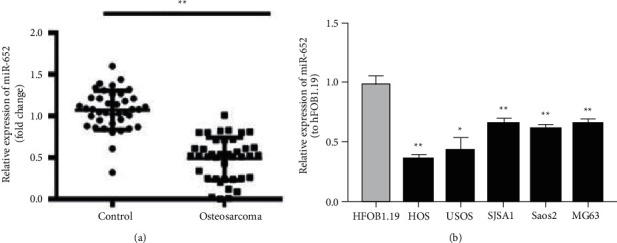
The expression of miR-652 in osteosarcoma tissues and cell lines. (a) The expression of miR-652 in osteosarcoma tissues. (b) The expression of miR-652 in osteosarcoma cell lines. ^*∗*^*P* < 0.05, ^*∗∗*^*P* < 0.01.

The expression of miR-652 in osteosarcoma tissue cell lines was detected by an RT-qPCR assay. The results showed that the mRNA expression level of miR-652 in osteosarcoma tissue was significantly lower than that of normal tissue (Figure 1(a)). The mRNA expression level of miR-652 in OS cell lines was significantly lower than that of normal osteoblasts. The expression of miR-652 was the lowest in HOS and MG63 (Figure 1(b)), and HOS and MG63 were used in follow-up experiments.

### 3.2. Overexpression of miR-652 Can Inhibit the Proliferation, Invasion, and Migration of Osteosarcoma Cells

**Figure 2:**
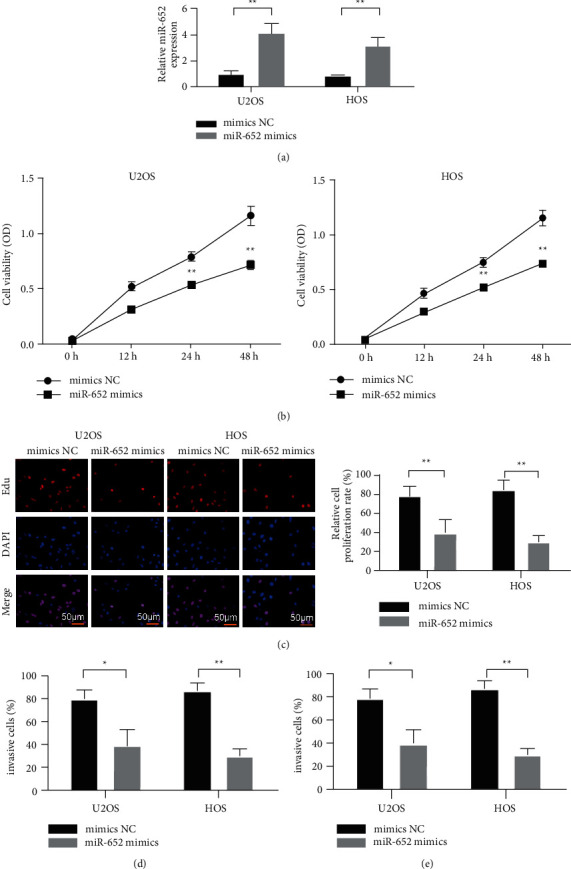
Overexpression of miR-652 inhibits the proliferation, invasion, and migration of osteosarcoma cells. (a) The expression of miR-652 detected by RT-qPCR. (b) Overexpression of miR-652 inhibits the viability of osteosarcoma cells. (c) Overexpression of miR-652 inhibits the proliferation of osteosarcoma cells (colonization). (d) Overexpression of miR-652 inhibits the invasion of osteosarcoma cells. (e) Overexpression of miR-652 inhibits the migration of osteosarcoma cells. ^*∗*^*P* < 0.05, ^*∗∗*^*P* < 0.01.

In order to explore the role of miR-652 in osteosarcoma cells, we transfected mimics NC and miR-652 mimics into HOS and MG63, respectively. The RT-qPCR assay showed that the expression of miR-652 was significantly increased in the miR-652 mimics group (Figure 2(a)). The CCK-8 assay showed that overexpression of miR-652 inhibited the viability of HOS and MG63 (Figures 2(b) and 2(c)). The results of the EDU assay showed that overexpression of miR-652 inhibited the proliferation of HOS and MG63 cells. In addition, cell invasion assays showed that overexpression of miR-652 inhibited the invasion ability of HOS and MG63 (Figures 2(d) and 2(e)).

### 3.3. HOXA9 Is a Potential Target Gene of miR-652

**Figure 3:**
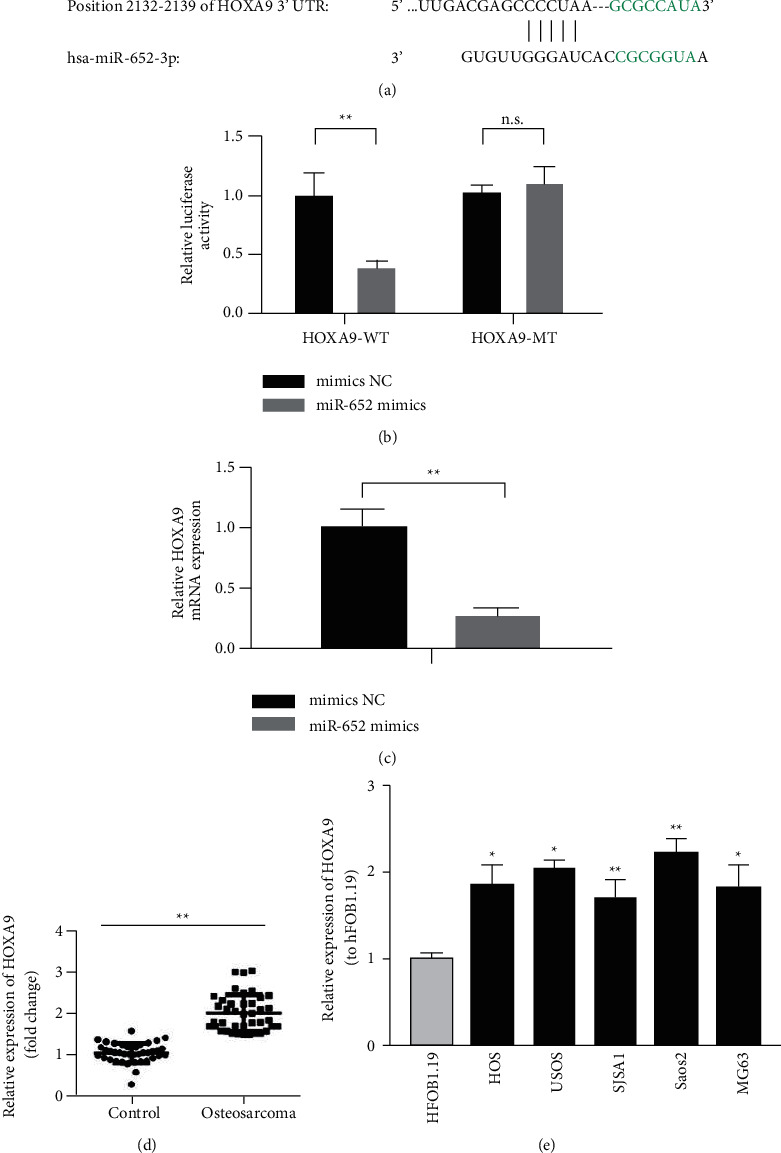
HOXA9 is a potential target gene of miR-652. (a) Predicting site between miR652 and HOXA9. (b) Targeting relationship between miR-652 and HOXA9 detected by dual-luciferase reporter gene assay. (c) The expression of HOXA9 detected by RT-qPCR. (d) The expression of HOXA9 was increased in osteosarcoma. (e) The expression of HOXA9 was increased in OS cell lines. ^*∗∗*^*P* < 0.01.

We predict that HOXA9 is a potential target gene of miR-652 through the TargetScan online tool and that there is a miR-652 binding site (Figure 3(a)) in the 3′UTR of HOXA9 mRNA. Dual-luciferase reporter gene assay further verified that miR-652 targets HOXA9 (Figure 3(b)). In addition, miR-652 mimics inhibited the expression of HOXA9 (Figure 3(c)). The results of the RT-qPCR assay showed that the mRNA expression level of HOXA9 in osteosarcoma tissues was significantly higher than that of normal tissue, and the mRNA expression level of HOXA9 in osteosarcoma cell lines was significantly higher than that of normal osteoblasts (Figures 3(d) and 3(e)).

### 3.4. HOXA9 Can Reverse the Effect of miR-652 on Osteosarcoma Cells

**Figure 4:**
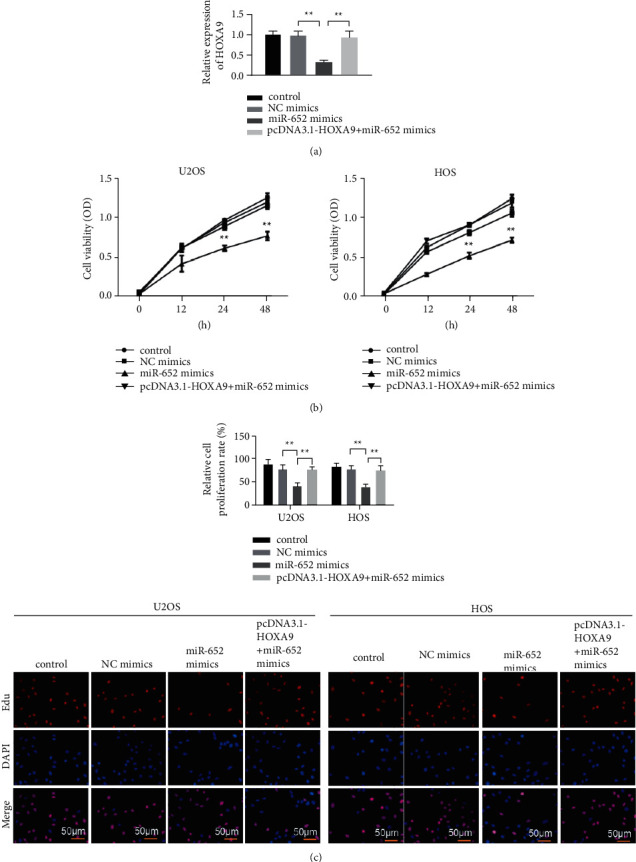
HOXA9 can reverse the effect of miR-652 on the proliferation of osteosarcoma cells. (a) The expression of HOXA9 detected by RT-qPCR. (b) The cell viabilities of U20S and HOS cells detected by CCK-8 assay. (c) EDU assay was used to detect the proliferation ability of U20S and HOS cells. ^*∗∗*^*P* < 0.01.

**Figure 5:**
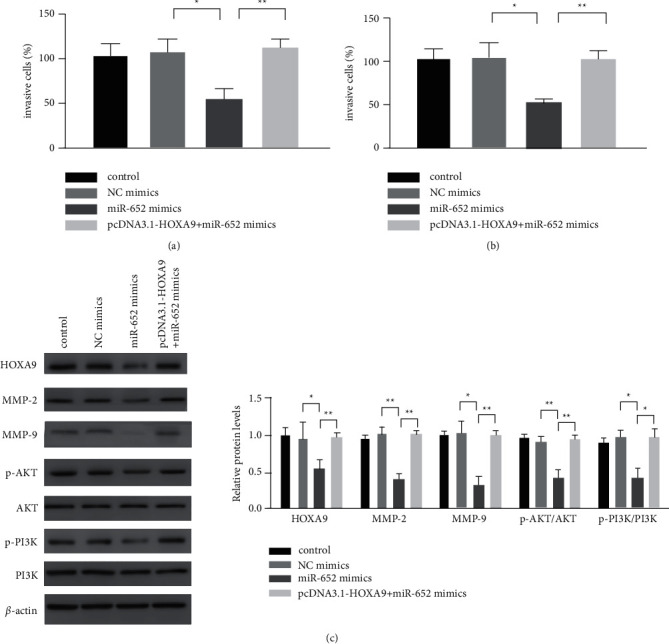
HOXA9 can reverse the effect of miR-652 on the invasion and migration of osteosarcoma cells. (a, b) The invasion and migration abilities of U20S and HOS cells detected by Transwell assay. (c) The expression of proteins detected by western blot assay. ^*∗*^*P* < 0.05, ^*∗∗*^*P* < 0.01.

In order to further verify the relationship between miR-652 and HOXA9, we set the control group, the NC mimics group, the miR-652 mimics group, and the pcDNA3.1HOXA9 + miR-652 mimics group in HOS and MG63, respectively. The RT-qPCR assay was used to detect the mRNA expression of HOXA9, and the results showed that the transfection was successful (Figure 4(a)). The results of the CCK-8 assay showed that the cell viability of the miR-652 mimics group was significantly lower than that of the NC mimics group, and the overexpression of HOXA9 reversed the effect of miR-652 mimics on cell viability (Figure 4(b)). Similarly, the results of the EDU assay showed that miR-652 mimics inhibited the proliferation of HOS and MG63 cells, while the overexpression of HOXA9 reversed the effect of miR-652 mimics on cell proliferation (Figure 4(c)). Then, we used the Transwell assay to detect the effect of miR-652 on cell invasion and migration. We found that miR-652 mimics significantly inhibited the ability of cell invasion and migration in HOS and MG63 compared with the NC mimics group. The overexpression of HOXA9 reversed the effect of miR-652 mimics on cell invasion and migration (Figures 5(a) and 5(b)). These results suggest that HOXA9 can reverse the proliferation, invasion, and migration of osteosarcoma cells induced by miR-652. In addition, we used a western blot assay to detect the expression of proteins related to the PI3K/Akt signaling pathway, and we found that miR-652 mimics significantly inhibited the expression of MMP-2, MMP-9, p-AKT, and p-PI3K compared with that of the NC mimics group. The expression of MMP-2, MMP-9, p-AKT, and p-PI3K in pcDNA3. 1HOXA9 and miR-652 mimics cotransfection group was significantly higher than miR-652 mimics group (Figure 5(c)).

## 4. Discussion

Osteosarcoma (OS) is a malignant tumor originating from mesenchymal tissue. It is the most common primary sarcoma of bone in children and young people [11]. Due to the characteristics of early metastasis of osteosarcoma, 80% of osteosarcoma patients suffer from metastatic or micrometastatic diseases at the time of diagnosis [12]. The most common cause of death in osteosarcoma is that tumor cells can break through the bone cortex and medullary cavity and transfer with blood to other tissues, especially the lungs [13]. Although medical technology has made great progress in recent years, the 5-year survival rate is higher than before, the overall survival rate is still very low [14]. Looking for tumor markers of osteosarcoma to help with its early diagnosis and treatment has become the focus of current research.

Microribonucleic acid (microRNA or miRNAs) is a kind of small noncoding single-stranded RNA widely expressed in organisms, which is about 18 to 25 bases in length. It can inhibit the expression of protein-coding genes at the post-transcriptional level by binding to the target mRNA [15]. Each miRNA has more than 1000 target genes, and each protein-coding gene can be regulated by multiple miRNAs. About 2603 miRNA have been identified (miRBASE, release21, 2014) in humans, which can regulate at least 60% of mRNA [16]. At present, the role of miRNA in tumors has been widely reported [17–20].

In recent years, it has been found that a variety of miRNA are abnormally expressed in osteosarcoma cells. The ectopic expression of these miRNAs affects the primary tumor growth, metastasis, drug resistance, and invasion of human OS cells, which play an important role in the occurrence and development of osteosarcoma. Xiao et al. [21] found that the level of miR-187 in OS tissues and cell lines decreased, and downregulation of S100A4 can inhibit the growth and metastasis of OS cells, which may be a potential biomarker and therapeutic target of OS. Yong et al. [22] proved that miR-613 was significantly downregulated in osteosarcoma patients, and the increased expression of miR-613 directly inhibited the expression of CXCR4, thus reducing the proliferation, migration, and apoptosis of osteosarcoma cells, as well as lung metastasis.

Previous studies have reported abnormal expression of miR-652 in a variety of cancers. miR-652 can activate the Wnt/*β*-catenin signaling pathway to promote the proliferation and metastasis of endometrial cancer cells by directly targeting RORA [23]. Sun et al. reported that miR-652 affects the progression of endometrial cancer by regulating RORA [23]. In lung cancer, Lgl1 was identified as the target gene of miR-652 [24]. In pancreatic cancer, miR-652 inhibits epithelial-mesenchymal transition by targeting ZEB1. In addition, miR-652-3p inhibition and decreased atherosclerosis by promoting cyclin D2 expression. However, the mechanism of miR-652 in the occurrence and development of osteosarcoma has not been reported in detail.

The abnormal expression of miR-652 in osteosarcoma and its related mechanism were discussed in this study. The results of the RT-qPCR assay showed that miR-652 was significantly downregulated in osteosarcoma tissues, and then the target gene interacting with miR-652 was predicted by bioinformatics to be HOXA9. The prediction hypothesis was further verified by a dual-luciferase reporter gene assay, which proved that miR-652 could indeed bind to the 3′-UTR region of HOXA9 mRNA. The effects of overexpression of miR-652 on the proliferation, migration, and invasion of U-2OS cells were observed. The results showed that the overexpression of miR-652 significantly decreased the ability of proliferation, migration, and invasion of U-2OS cells, while overexpression of the target gene HOXA9 reversed these conditions. It is inferred that miR-652 regulates the proliferation, migration, and invasion of osteosarcoma cells by targeting HOXA9. The HOXA9 gene is a member of the homeobox (HOX) gene family. Its encoding product is an important transcription regulator. HOXA9 plays an important role in controlling embryonic development and regulating cell differentiation. Recent studies have found that the abnormal expression of the HOXA9 gene is closely related to acute leukemia, glioblastoma, ovarian cancer, lung cancer, breast cancer, and other tumors. HOXA9 has dual effects (procancer or anticancer) by participating in the proliferation, apoptosis, or differentiation process of tumor cells in different tumor types or at different stages of tumor. The exact mechanism for this difference is unclear.

PI3K/Akt is a classic signaling pathway, which regulates cell proliferation, apoptosis, differentiation, migration, and invasion, and is also involved in the occurrence and development of many cancers [25–27]. However, it is not clear whether miR-652 can affect the biological behavior of osteosarcoma cells by regulating the PI3K/Akt signal pathway. The purpose of this study was to observe the changes of the PI3K/Akt signal pathway related to HOXA9 in miR-652 from the level of protein expression. The results showed that overexpression of miR-652 could inhibit the protein expression of downstream invasion and migration-related molecules MMP2 and MMP9 caused by the activation of this pathway, thus inhibiting cell migration and invasion, and the overexpression of HOXA9 could reverse this situation and inhibit cell migration and invasion. It can be concluded that miR-652 negatively regulates the expression of genes in osteosarcoma cells, inhibits the activation of the PI3K/Akt signaling pathway, and further inhibits the migration and invasion of cancer cells.

There are also some deficiencies in this study. Firstly, this study demonstrated the antitumor effect and molecular mechanism of miR-652 through cell experiments. However, *in vivo* animal models to demonstrate the anticancer effect of miR-652 are lacking. Secondly, the upstream regulatory factors of miR-652 were also not involved in this study. In addition to HOXA9, whether there are other target genes also needs to be further studied.

## 5. Conclusions

The expression of miR-652 is low in osteosarcoma, and miR-652 inhibits the viability and migration of osteosarcoma cells by regulating HOXA9. Therefore, miR-652 may act as a tumor suppressor gene in osteosarcoma and provide a new target for molecular therapy of osteosarcoma.

## Data Availability

The analyzed data sets generated during the study are available from the corresponding author on reasonable request.
